# Increased Frequencies of Th22 Cells as well as Th17 Cells in the Peripheral Blood of Patients with Ankylosing Spondylitis and Rheumatoid Arthritis

**DOI:** 10.1371/journal.pone.0031000

**Published:** 2012-04-02

**Authors:** Lei Zhang, Yong-gang Li, Yu-hua Li, Lei Qi, Xin-guang Liu, Cun-zhong Yuan, Nai-wen Hu, Dao-xin Ma, Zhen-feng Li, Qiang Yang, Wei Li, Jian-min Li

**Affiliations:** 1 Department of Orthopedics, Qilu Hospital, Shandong University, Jinan, China; 2 Department of Emergency, Qilu Hospital, Shandong University, Jinan, China; 3 Department of Hematology, Qilu Hospital, Shandong University, Jinan, China; 4 Department of Obstetrics and Gynecology, Qilu Hospital, Shandong University, Jinan, China; 5 Department of Rheumatology, Provincial Hospital affiliated to Shandong University, Jinan, China; 6 Department of Clinical Laboratory, Qilu Hospital, Shandong University, Jinan, China; University Medical Center Freiburg, Germany

## Abstract

**Background:**

T-helper (Th) 22 is involved in the pathogenesis of inflammatory diseases. The roles of Th22 cells in the pathophysiological of ankylosing spondylitis (AS) and rheumatoid arthritis (RA) remain unsettled. So we examined the frequencies of Th22 cells, Th17 cells and Th1 cells in peripheral blood (PB) from patients with AS and patients with RA compared with both healthy controls as well as patients with osteoarthritis.

**Design and Methods:**

We studied 32 AS patients, 20 RA patients, 10 OA patients and 20 healthy controls. The expression of IL-22, IL-17 and IFN-γ were examined in AS, RA, OA patients and healthy controls by flow cytometry. Plasma IL-22 and IL-17 levels were examined by enzyme-linked immunosorbent assay.

**Results:**

Th22 cells, Th17 cells and interleukin-22 were significantly elevated in AS and RA patients compared with OA patients and healthy controls. Moreover, Th22 cells showed positive correlation with Th17 cells as well as interleukin-22 in AS and RA patients. However, positive correlation between IL-22 and Th17 cells was only found in AS patients not in RA patients. In addition, the percentages of both Th22 cells and Th17 cells correlated positively with disease activity only in RA patients not in AS patients.

**Conclusions:**

The frequencies of both Th22 cells and Th17 cells were elevated in PB from patients with AS and patients with RA. These findings suggest that Th22 cells and Th17 cells may be implicated in the pathogenesis of AS and RA, and Th22 cells and Th17 cells may be reasonable cellular targets for therapeutic intervention.

## Introduction

Ankylosing spondylitis (AS) is a chronic inammatory disease that is characterized by mainly involving bilateral sacroiliitis and axial joints, but sometimes peripheral joints and extra-articular organs are also involved [1]. Rheumatoid arthritis (RA), which represents an example of autoimmunity disease, is another form of arthritis. The abnormality of T cells is implicated in the pathogenesis of many autoimmune diseases, and many autoimmune diseases, especially arthritis, were considered to be mainly driven by Th1 cells [1]–[3]. A new IL-17-producing T cell subset, termed Th17 cells, has been described in recent years [4]–[7]. It has been established that Th17 cells play critical roles in several animal models of autoimmunity, such as experimental allergic encephalomyelitis (EAE) [8] and murine arthritis models [9], [10]. Besides, Th17 cells are considered to be involved in many human inflammatory diseases, including multiple sclerosis, psoriasis and inflammatory arthritis [11]–[15]. As to AS and RA, increased Th17 cells were found in PBMC from patients with AS and RA [16]. IL-17, secreted mainly by Th17 cells, is a cytokine shown to stimulate RA synovial fibroblast (RASF) to release several mediators of joint inflammation including IL-6, IL-8, GM-CSF and PGE2 [17]–[19]. Moreover, elevated serum levels of IL-17 and IL-23 has been reported in AS which is one of the forms of arthritis [20].

IL-22, a member of IL-10 cytokine family, exerts its effects via a heterodimeric transmembrane receptor complex consisting of IL-10R2 and IL-22R1 [21]. IL-22 has been believed as an important player in regulating inflammatory responses associated with many inflammatory diseases. Higher expression of IL-22 mRNA was observed in psoriatic skin lesion, and elevated serum IL-22 levels were found in patients with psoriasis [22]. In addition, the involvement of IL-22 in other inflammatory diseases such as inflammatory bowel disease [23] also proves its proinflammatory roles. However, diminishing intestinal inflammatory in a mouse model of ulcerative colitis and providing protection to hepatocytes during acute liver inflammation by IL-22 demonstrate its anti-inflammatory properties [24]. The situation of IL-22 was not completely consistent in autoimmune diseases. Consistent with psoriasis, increased IL-22 has also been found in serum samples from RA and Crohn disease patients. On the contrary, decreased plasma IL-22 levels were found in patients with SLE [25]. So, diverse pathogenic mechanisms and tissue microenvironments may result in different contributions of IL-22 in autoimmune disease development. The precise pathophysiologic function of IL-22 remains unclear, and the involvement of IL-22 in AS and RA remains to be established. 

Th22 subset is a more recently identified new human T helper subset, which is characterized by abundant secretion of IL-22 but not IL-17 or IFN-γ [26]–[28]. Th22 cells express the chemokine receptors CCR4, CCR6 and CCR10 [26]. Moreover, this newly identified CD4^+^ T cells clones have low or undetectable expression of Th1 and Th17 transcription factor T-bet and RORγt, and arylhydrocarbon receptor (AHR) has been considered to be the key transcription factor of Th22 subset [26]. In addition, naïve T cells differentiate toward the Th22 phneotype in the presence of IL-6 and TNF-α[26]. All of above provide strong evidence that Th22 cells represent an independent and terminally differentiated T cells subtype. It has been reported that Th22 cells were detected in psoriatic skin lesions. Moreover, the increasing circulating Th22 cells [29] suggest that Th22 cells may be implicated in the pathogenesis of psoriasis which is a chronic inammatory disease. In addition, elevated Th22 cells in peripheral blood of RA patients have also been reported in our previous study. Thus, the involvement of Th22 cells in other chronic inammatory diseases needs to be further investigated.

The roles of both Th22 cells and IL-22 in the pathogenesis of ankylosing spondylitis and rheumatoid arthritis are still unclear and remain to be clarified. Therefore, to investigate their roles in the pathogenesis of AS and RA, we examined the frequencies of Th22 cells in peripheral blood as well as the levels of plasma IL-22 of both AS and RA patients, and assayed their correlations with disease activity in this study.

## Materials and Methods

### Ethics Statement

Enrollment took place between May, 2010 and April, 2011 in two centers: Qilu Hospital, Shandong University and Shandong Provincial Hospital, Shandong University, China. Our research has been approved by both the Medical Ethical Committee of Qilu Hospital, Shandong University and the Medical Ethical Committee of Shandong Provincial Hospital, Shandong University. A written informed consent document has been obtained from each participant.

### Patients and Controls

A total of 32 patients with AS according to the modified New York criteria [30] ere recruited in this study. The Bath Ankylosing Spondylitis Disease Activity Index (BASDAI) score [31] were measured for the patients with AS. All patients were HLA-B27-positive. This group consisted of 27 men and 5 women, with mean ± SD disease duration of 8.6±5.9 years. The mean age of the patients was 36.6±10.2 years. A total of 20 patients with active RA according to the criteria of the American College of Rheumatology were included in this study [32]. The DAS28 score [33] were measured for the patients with RA. This group consisted of 16 women and 4 men, with mean ± SD disease duration of 8.9±3.9 years. The mean age of the patients was 47.5±9.2 years. The demographic and key clinical information of AS and RA patients are summarized in Table 1 and Table 2. All of the patients did not receive immunosuppressive or immunomodulatory drugs for at least 2 months when sampling. The major previous treatment of AS and RA patients were shown in Table S1 and Table S2. Ten osteoarthritis (OA) patients (3 females and 7 males; mean age 48.9±10.3 years) as disease controls and twenty healthy controls (5 females and 15 males; mean age 37.9±9.1 years) were also recruited in the study, and all of them did not have any rheumatologic conditions.

**Table 1:** Characteristics of the patients with Ankylosing Spondylitis.*

Characteristics	value
Age(y)	36.6±10.2
Sex(male/female)	27/5
Disease duration(y)	8.6±5.9
ESR(mm/h)	38.2±20.8
ESR(mm/h) of Healthy Control	9.5±4.2
CRP(mg/L)	24.9±13.7
CRP(mg/L) of Healthy Control	3.4±1.6
BASDAI score	3.2±1.7
HLA-B27,positive member	32

* BASDAI = Bath Ankylosing Spondylitis Disease Activity Index (range 0–10); ESR  = erythrocyte sedimentation rate; CRP = C-reactive protein.

**Table 2:** Demographic and clinical characteristics of RA patients.*

Characteristics	value
No. of patients	20
Age(y)	47.5±9.2
Sex(male/female)	4/16
Disease duration(y)	8.9±3.9
RF	14/20(70%)
ESR(mm/h)	35.8±27.9
CRP(mg/L)	27.1±23.3
No. of swollen joints	7.3±3.7
No. of tender joints	8.1±3.8
DAS28	5.2±1.4

* RF = rheumatoid factor; ESR = erythrocyte sedimentation rate; CRP = C-reactive protein; DAS28 = Disease Activity Score in 28 joints.

### Flow Cytometric Analysis

Intracellular cytokines were studied by flow cytometry to reflex the cytokine-producing cells. Briefly, heparinized peripheral whole blood (400 µl) with an equal volume of Roswell Park Memorial Institute 1640 medium were incubated for 4 h at 37°C, 5% CO2 in the presence of 25 ng/mL of phorbol myristate acetate (PMA), 1 µg/mL of ionomycin, and 1.7 µg/ml Golgiplug(Monensin; all from Alexis Biochemicals, San Diego, CA, USA). PMA and ionomycin are pharmacological T-cell-activating agents that mimic signals generated by the T-cell receptor (TCR) complex and have the advantage of stimulating T cells of any antigen specificity. Monensin was used to block intracellular transport mechanisms, thereby leading to an accumulation of cytokines in the cells. After incubation, the cells were stained with PE-Cy5-conjugated anti-CD4 monoclonal antibodies (clone: RPA-T4, Cat: 45-0049-42) at room temperature in the dark for 20 min. The cells were next stained with FITC-conjugated anti-interferon (IFN)-γ monoclonal antibodies (clone: 4S-BS, Cat: 11-7319-82), PE-conjugated anti-IL-17A monoclonal antibodies (clone: eBio64DEC17, Cat: 12-7179-42) and APC-conjugated anti-IL22 monoclonal antibodies (clone: 22URTI, Cat: 50-7229-42) after fixation and permeabilization. All the antibodies were from eBioscience, San Diego, CA, USA. Isotype controls were given to enable correct compensation and confirm antibody specificity. Stained cells were analyzed by flow cytometric analysis using a FACScan cytometer equipped with CellQuest software (BD Bioscience PharMingen). Th22, Th17, Th1 and Th1/Th17 cells were defined as CD4^+^IFNγ^–^IL17^–^IL-22^+^, CD4^+^IFNγ^–^IL17^+^, CD4^+^IFNγ^+^ and CD4^+^IFNγ^+^IL17^+^T cells respectively.

### IL-22 and IL-17 Enzyme-linked Immunosorbent Assay (ELISA)

Peripheral blood was collected into heparin-anticoagulant vacetainer tubes. Plasma was obtained from all subjects by centrifugation and stored at –80°C for determination of cytokines. Plasma IL-22 (Cat: BMS2047) and IL-17 (Cat: BMS2017) levels were determined with a quantitative sandwich enzyme immunoassay technique in accordance with the manufacturer’s recommendations (lower detection limit 9 pg/ml; eBioscience).

### Clinical Assessment

BASDAI score of AS patients and disease activity score in 28-joints (DAS28) of RA patients was calculated in our study. At the time of clinical assessment for disease activity, blood samples were collected for the measurement of levels of C-reactive protein (CRP) and ESR.

### Statistical Analysis

Results were expressed as mean ± SD or median (range). Statistical significance of Th22, Th17, Th1 and plasma IL-22 as well as IL-17 among patients with AS, RA, OA and HC was determined by ANOVA, and difference between two groups was determined by Newman–Keuls multiple comparison test (*q* test) unless the data were not normally distributed, in which case Kruskal - Wallis test (*H* test) and Nemenyi test were used. The Pearson or Spearman correlation test was used for correlation analysis depending on data distribution. All tests were performed by SPSS 17.0 system. P value less than 0.05 was considered statistically significant.

## Results

### Elevated Th22 Cells Correlated with Increased Plasma Levels of IL-22 in AS and RA Patients

**Figure 1:**
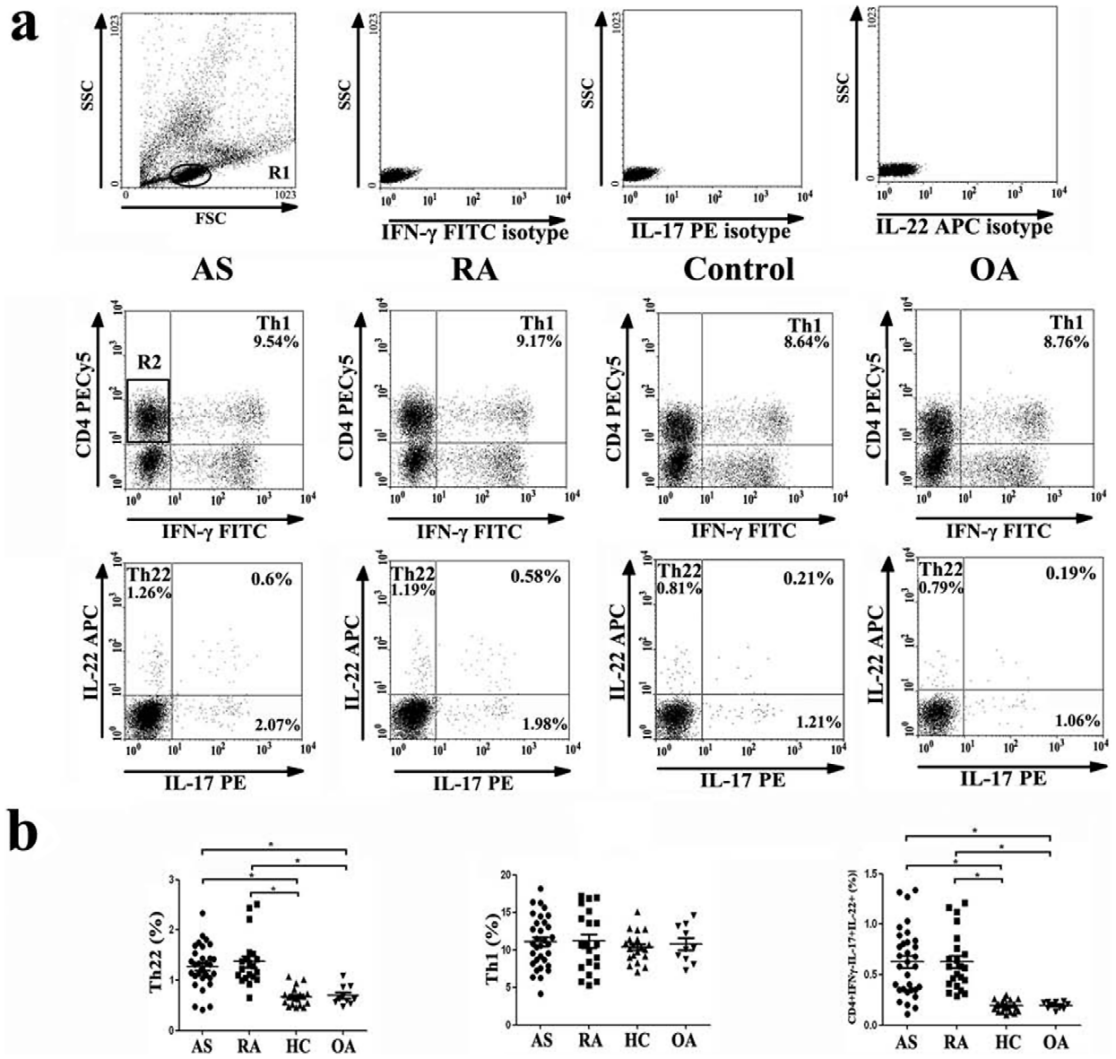
Circulating Th22 cells and CD4^+^IFNγ^-^IL17^+^IL-22^+^ T cells are significantly increased in ankylosing spondylitis (AS) patients and rheumatoid arthritis (RA) patients compared with osteoarthritis (OA) patients and healthy controls. **a,** Representative flow cytometry dot plots example of each group. **b,** The percentages of circulating Th22 cells (left panel), Th1 cells (middle panel) and CD4^+^IFNγ^-^IL17^+^IL-22^+^ T cells (right panel) from AS, RA, OA patients and healthy controls after stimulation with phorbol myristate acetate, ionomycin, and monensin for 4 h. (* =  *P*<0.05).

We analyzed the frequency of Th22 cells based on cytokine patterns after in vitro activation by PMA/ionomycin in short-term cultures. The expression of a typical dot-plot of Th22 cells in representative AS, RA as well as OA patients and healthy controls was shown in Fig. 1a. The percentage of Th22 cells was significantly elevated in AS (1.27±0.42%) and RA (1.37±0.49%) patients compared to OA patients (0.70±0.19%) or healthy controls (0.68±0.18%) (Fig. 1b). 

In addition, we also quantified the number of Th22 cells per volume (50µL) of peripheral blood. The number of Th22 cells was significantly increased in AS (204±34) and RA (211±43) patients compared with healthy controls (123±23) after stimulation with phorbol myristate acetate, ionomycin, and monensin for 4 h (Fig. S2a).

**Figure 2:**
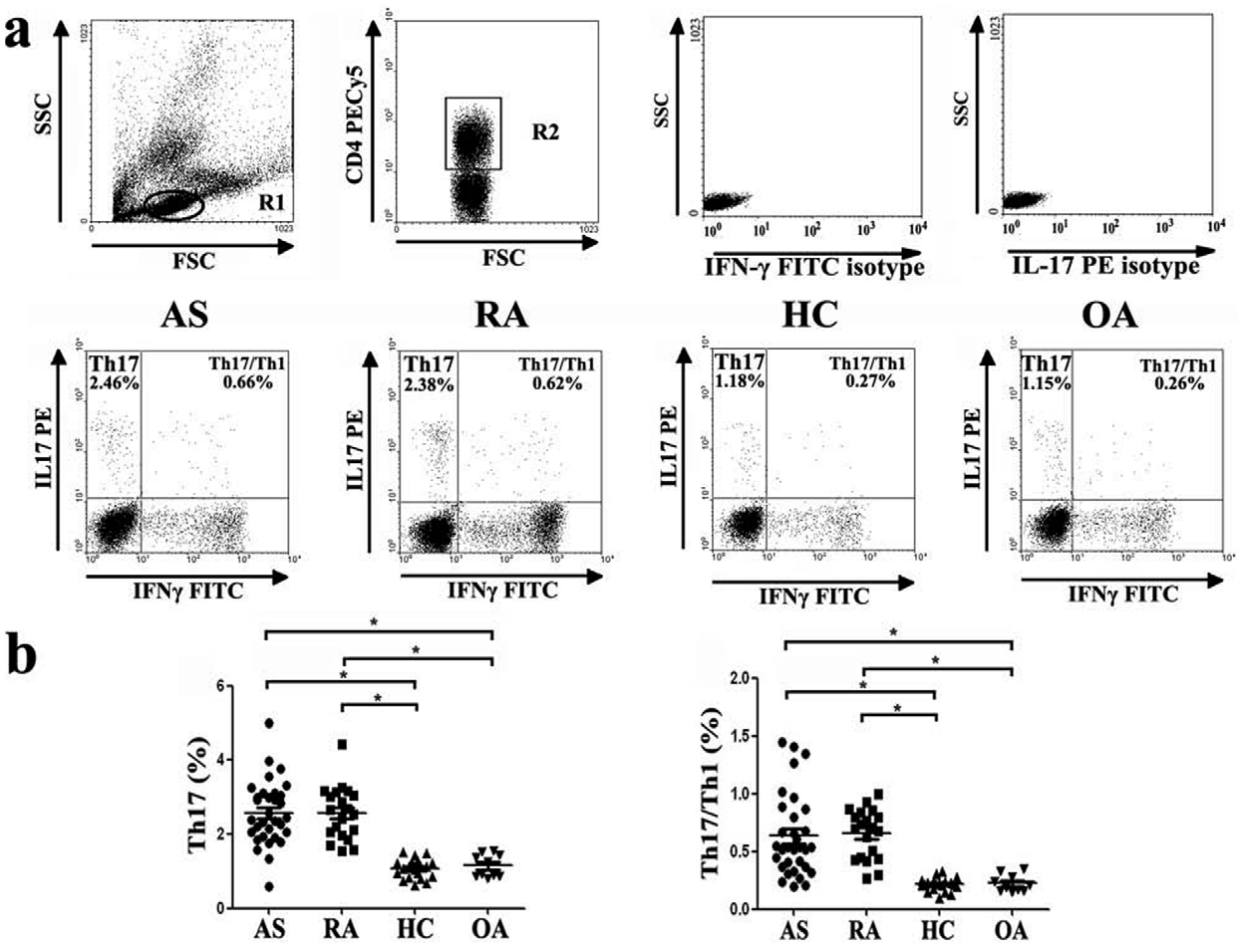
Concentrations of IL-22 and IL-17 in plasma of non-stimulated peripheral blood from AS, RA, OA patients and healthy controls. **a,** Concentrations of IL-22 in plasma from AS, RA, OA patients and healthy controls. The levels of IL-22 was significantly increased in AS and RA patients compared with OA patients or healthy controls. **b,** Concentrations of IL-17 in plasma from AS, RA, OA patients and healthy controls. As to IL-17, there was no significant difference between AS or RA patients and OA as well as healthy controls (* =  *P*<0.05).

Plasma levels of IL-22 were examined by ELISA. The levels of IL-22 were significantly increased in AS (41.03±16.00pg/ml) and RA (44.53±29.84pg/ml) patients compared to OA patients (24.53±3.45pg/ml) and healthy controls (25.33±3.75pg/ml) (Fig. 2a).

**Figure 3:**
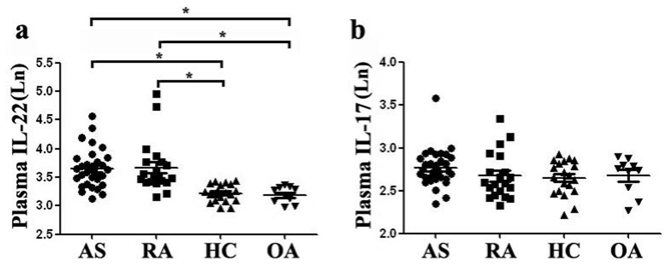
Correlation between the percentages of each T cell subset and the plasma IL-22 concentrations in AS or RA patients. **a and b**, Positive correlation was found between Th22 cells and IL-22 in AS (**a**) and RA (**b**) patients. **c,** Positive correlation was found between Th17 cells and IL-22 in AS patients.

A positive correlation was found between Th22 cells and plasma levels of IL-22 in AS (r = 0.743, *P*<0.001; Fig. 3a) and RA (r = 0.548, *P* = 0.027; Fig. 3b) patients. Moreover, positive correlations were also found between IL-22 plasma levels and Th17 cells (r = 0.587, *P* = 0.045; Fig. 3c) in AS patients. However, no corresponding correlation was found in RA patients (*P* = 0.801) (Fig. S1l).

### Elevated Th17 Cells in AS and RA Patients

**Figure 4:**
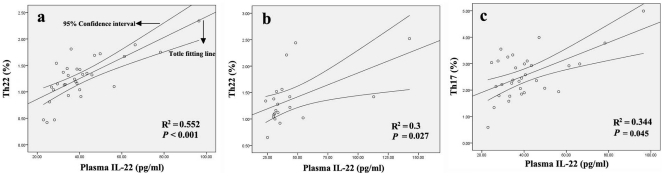
Circulating Th17 cells and Th17/Th1 cells are significantly increased in ankylosing spondylitis (AS) patients and rheumatoid arthritis (RA) patients compared with osteoarthritis (OA) patients and healthy controls. **a,** Representative flow cytometry dot plots example of each group. **b,** The percentages of circulating Th17 cells (left panel) and Th17/Th1 cells (right panel) from AS, RA, OA patients and healthy controls after stimulation with phorbol myristate acetate, ionomycin, and monensin for 4 h (* =  *P*<0.05).

The expression of a typical dot-plot of Th17 cells in representative AS, RA, OA patients and healthy controls was shown in Fig. 4a. We found significantly increased percentage of Th17 cells in AS (2.58±0.86%) and RA (2.57±0.72%) patients compared with OA patients (1.15±0.31%) and healthy controls (1.07±0.26%)(Fig. 4b). Consistently, the number of Th17 cells per volume (50µL) of peripheral blood was significantly increased in AS (320±36) and RA (337±39) patients compared with healthy controls (214±26) after stimulation with phorbol myristate acetate, ionomycin, and monensin for 4 h (Fig. S2b). However, there was no significant difference regarding plasma IL-17 between each group. (AS: 16.28±4.25, *P*>0.05; RA: 15.02±4.60, *P*>0.05; OA: 14.80±2.88, *P*>0.05; HC: 14.39±2.72, *P*>0.05) (Fig. 2b).

As to Th1 cells, there was no significant difference between each group. (AS: 11.05±3.41%, *P*>0.05; RA: 11.13±4.09%, *P*>0.05; OA: 10.73±2.50%, *P*>0.05; HC: 10.37±2.00, *P*>0.05) (Fig. 1a, b). 

### Increased Expression of IL-17 and IL-22 Double-Positive CD4 T Cells as well as IL-17 and IFN-γ Double-Positive CD4 T Cells in AS and RA Patients

For these experiments, we also examined the frequencies of CD4^+^IFNγ^-^IL17^+^IL-22^+^ T cells and CD4^+^IFNγ^+^IL17^+^ T cells. Though most Th17 cells did not simultaneously express both IL-22 and IL-17, the percentage of CD4^+^IFNγ^-^IL17^+^IL-22^+^ T cells was significantly increased in AS (0.63±0.34%)) and RA (0.65±0.29%) patients compared with OA patients (0.20±0.04%) and healthy controls (0.19±0.05%)(Fig. 1b). In addition, ratios of Th1/Th17 cells were significantly increased in AS patients (0.63±0.35%) and RA patients (0.66±0.21%) compared to OA patients (0.22±0.07%) and healthy controls (0.22±0.06%). (Fig. 4b).

### Correlation between Th22, Th17 and Th1 Cells in AS and RA Patients

**Figure 5:**
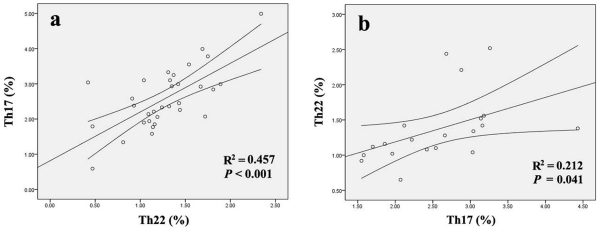
Correlation between the percentages of Th22 cells and the percentages of Th17 cells in AS and RA patients. **a,** Positive correlation was found between Th22 cells and Th17 cells in AS patients. **b,** Positive correlation was found between Th22 cells and Th17 cells in RA patients.

In AS patients, there was a significant positive correlation between Th22 cells and Th17 cells (r = 0.676, *P*<0.001) (Fig. 5a). Similarly, a positive correlation was also found between Th22 cells and Th17 cells (r = 0.46, *P* = 0.041) in RA patients (Fig. 5b). However, Th1 cells failed to show a significant correlation with Th22 cells and Th17 cells.

### The Correlation of the Frequencies of Th22 Cells and Th17 Cells with Disease Activity or Laboratory Parameters in AS and RA Patients

**Figure 6:**
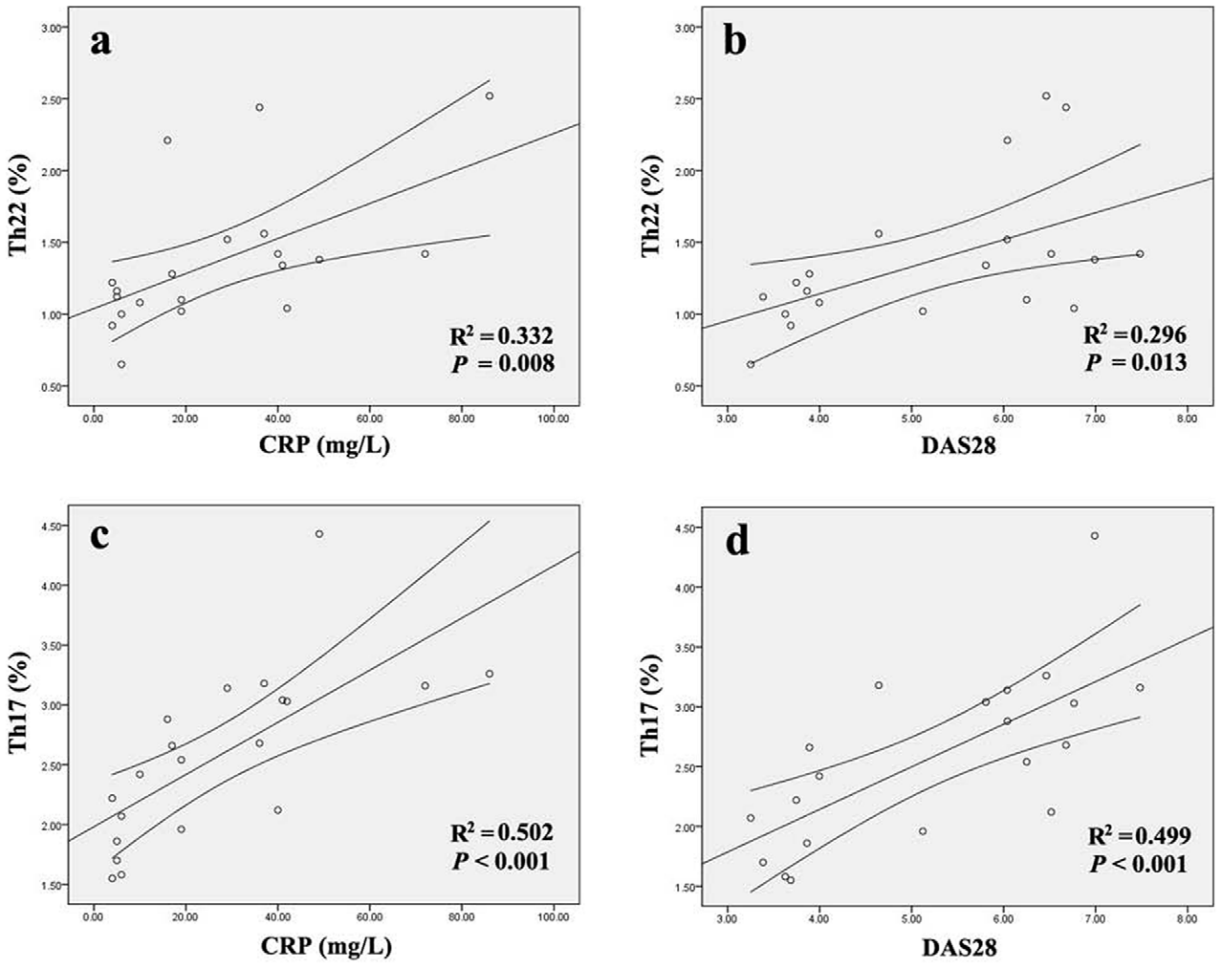
Correlation between the percentages of Th22 cells as well as Th17 cells and CRP levels as well as DAS28.

In patients with RA, there were positive correlations between the percentage of Th22 cells and CRP level or DAS28 (r = 0.576, *P*  = 0.008 or r = 0.544, *P*  = 0.013 respectively) (Fig. 6 a, b). Consistently, positive correlations were also found between the percentage of Th17 cells (r = 0.709, *P*<0.001 or r  = 0.706, *P*<0.001 respectively) (Fig. 6 c, d) and CRP level as well as DAS28. However, the percentage of Th1 cells was not correlated with either CRP level or DAS28 (*P*  = 0.105 or *P*  = 0.205), and plasma level of IL-22 or IL-17 failed to show a statistical correlation with CRP level or DAS28 (*P* = 0.622 and *P*  = 0.357 or *P* = 0.317 and *P* = 0.872) in RA patients (Fig. S1j, k). In patients with AS, there was no correlation between the percentage of Th22 cells and clinical parameters, including ESR (*P* = 0.964), CRP (*P* = 0.393) and BASDAI score (*P* = 0.226) (Fig. S1a, b, c). Consistently, no correlation was found between plasma level of IL-22 and clinical parameters in AS patients, including ESR (*P* = 0.918), CRP (*P* = 0.862) and BASDAI score (*P* = 0.320) (Fig. S1g, h, i). Correlation analysis between the percentage of Th17 cells and clinical parameters also showed no association (*P* = 0.189, *P* = 0.852 and *P* = 0.733 respectively) (Fig. S1d, e, f).

## Discussion

Th22 cells, a recently defined lineage of T cells distinct from Th1, Th2 and Th17 cells, have been believed to play a complicated and important role in inflammatory and autoimmune diseases. The function of Th22 cells is achieved through the signature cytokine they secreted. Th17 cells have already been proposed to be the primary driver of autoimmune diseases, including rheumatoid arthritis. However, the potential role of Th22 cells in inflammatory arthritis such as AS and RA is far from clear.

To determine whether Th22 is involved in AS and RA, the percentages of Th22 cells were examined in the peripheral blood of patients with AS, RA OA, and healthy controls in this study. This is the first study that evaluates the relative abundance of Th22 cells in peripheral blood of RA and AS patients. Our results demonstrated that the percentages of Th22 cells were significantly elevated in the peripheral blood of patients with AS and also in those with RA compared with OA patients and healthy controls, which was consistent with our previous experiments on RA [34]. Moreover, we also quantified the number of Th22 cells in per volume blood. Consistent with the results of the percentage, the number of Th22 cells in per volume of blood was significantly increased in AS and RA patients compared to healthy controls. Likewise, increased Th22 cells has been observed in peripheral blood of patients with psoriasis [29], which implicating the involvement of Th22 cells in the chronic inflammatory skin disorder. These observations are compatible with the idea that Th22 cells may contribute to the pathogenesis of both RA and AS, which are two types of inflammatory arthritis. In the present study, correlation was also found between levels of Th22 cells and disease activity as assessed by DAS28 or the CRP concentration in RA. However, such correlation was not observed in AS. The possible explanation for this inconsistency between AS and RA is that many patients with AS do not have elevated CRP or ESR levels, so it is more difficult to assess disease activity in AS. It is known that Th17 cells are enriched in the joints of RA patients and co-express the chemokine receptor CCR6 and CCR4 [35]. Besides, CCL20 [36] as well as CCL22 [37], the ligand of CCR6 and CCR4 respectively, have already been observed in synovial fluid of human. Thus, all signs indicate that the interaction between receptor and ligand may play an important role in attracting Th17 cells to the joints in RA. Similar to Th17 cells, Th22 cells also co-express the chemokine receptor CCR6 and CCR4. So, we can reasonable believe that Th22 cells might selectively migrate to the joints from peripheral blood in the similar way. Moreover, we also speculated that Th22 cells might be elevated in the joints of RA and AS patients, which could play a pathogenic role in the lesions. Otherwise, what the pathogenic role does the Th22 cells play in AS and RA when migrating to the lesion remain to be elucidated. Then, we would focus our further investigation on the involvement of this new subset in synovial fluid of patients’ joints.

IL-22, a cytokine of IL-10 family, is the most important functional cytokine of Th22 cells. Considerable evidence suggests that IL-22 may be involved in the pathogenesis of many inflammatory diseases, but its pathophysiologic function is not well known. To investigate the potential role of IL-22 in AS and RA patients, we examined the concentrations of plasma IL-22 in our experiments. In line with our previous study, elevated levels of plasma IL-22 were also detected in AS and RA patients in the present study, implicating that IL-22 may be involved in the pathogenesis of both AS and RA. IL-22 has been demonstrated to exert its biological effects via binding to the heterodimeric receptor complex, including IL-22R1 and IL-10R2, and IL-22 activates Jak1 and Tyk2, which further activates STAT-1, STAT-3 and STAT-5 [38]. It has already been reported that IL-22R1 mRNA was expressed in RA synovial tissue. In addition, activation of ERK-1/2 as wells as p38 MAPK by IL-22 has also been reported in RA [39]. In vitro, recombinant IL-22 promoted proliferation of synovial fibroblasts of RA patients (RASF) and increased production of monocyte chemoattractant protein 1 (MCP-1) by RASF. Thus, we speculated the similar effect of IL-22 in AS patients as in RA patients. More recently, IL-22 serum levels were demonstrated to be associated with radiographic progression in rheumatoid arthritis [40], which further improved the importance of IL-22 in chronic inflammatory arthritis. In our study, plasma IL-22 levels correlated positively with Th22 cells in both AS patients and RA patients. In addition, correlation between plasma IL-22 level and Th17 cells were observed in AS patients. By contrast, no association of plasma IL-22 levels with Th17 cells was found in RA patients. Th22 cells, Th17 cells and Th1 cells are the main T cells subsets secreting IL22 [41], [26], [28], [42]. The correlation between Th17 cells and IL-22 in AS supported the idea that Th17 subset was an important T cells subset secreting IL-22 in peripheral blood of AS patients. Otherwise, no correlations in RA patients suggested that Th22 cells may produced much greater portion of total IL-22, and Th17 cells may produced a relatively smaller portion of total IL-22 in peripheral blood of RA patients than AS patients. So far, the pathophysiologic role of IL-22 in both AS and RA is not fully understood and need further investigation.

Considerable evidence suggests Th17 cells and Th1 cells have been involved in the development of autoimmune diseases [43], [44]. Thus, we examined the frequencies of Th17 cells and Th1 cells in each group in this experiment. In consistence with the experiment of Hui *et al* [16], the percentages of Th17 cells were demonstrated to be significantly increased in the peripheral blood of patients with AS and RA. Consistent with the results of Th22 cells, the number of Th17 cells in per volume of blood was significantly increased in AS and RA patients compared to healthy controls. In contrast to the results for Th17 and Th22 cell, no statistical difference was detected in percentages of Th1 cells between each group. Our observations supported the idea that Th17 cells contributed to the pathogenesis of AS and RA. Furthermore, we also examined the correlation between disease activity of AS or RA and the percentages Th17 cells. Like Th22 cells, there was a positive correlation between disease activity and Th17 in RA patients but not in AS patients. This inconsistent results between T cells and disease activity in AS is unclear, and needs further study. In this study, a positive correlation was found between the frequencies Th22 cells and Th17 cells in peripheral blood of patients with both AS and RA. This co-elevated level of Th22 cells and Th17 cells suggests these two T cell subsets may play a synergistic role in AS and RA, and the specific links between Th22 cells and Th17 cells need further investigation.

IL-17, the main cytokine of Th17 cells, contributes to the pathogenesis of arthritis as has been demonstrated in many experimental arthritis models. Although elevated levels of IL-17 have been observed in synovial fluid of RA patients, the levels of this T cell cytokine in plasma of these patients is hard to detect. Furthermore, the reports on IL-17 levels in plasma of RA or AS patients are inconsistent [35], [20]. The levels of IL-17 in plasma of each group were also measured in this experiment. In line with recent report [35], plasma IL-17 levels were not significantly different between the patients and healthy controls in our study. Moreover, the situation of IL-17 in autoimmune diseases is different. Increased levels of IL-17 have been shown in the serum of systemic sclerosis patients, but not in that of SLE patients or healthy controls.

In humans, it is not unusual to encounter dual +Th1/Th17 cells. It has previously been demonstrated that T cells from synovial fluid of RA can co-express IL-17 and IFN-γ [45], and both IL-17 and IFN-γ can be secreted by T cells derived from the joints [46]. In addition, IL-17 and IFN-γ cytokines have been shown to be co-expressed in human memory CD4+CD45+RO+ T cells from treatment-naïve early RA patients [47]. So we examined the percentages of Th17/Th1 cells. Consistent with a recent report in psoriasis [29], Th17/Th1 cells were increased in peripheral blood of AS and RA compared with OA and healthy controls. However, cells secreting IFN-γ alone were much more than those secreting IL-17 after stimulating with PMA and ionomycin; therefore, the contribution by Th17 cells to total IFN-γ secretion would be very low. Because Th17 cells also produced IL-22, Th17 cells that were positive for IL-22 were examined in this study. It has been shown that IL-22-positive Th17 cells were only observed in human but not in mouse [48]. In this experiment, increasing percentages of CD4+IL-22+IL-17+IFNγ- T cells were detected in peripheral blood from AS and RA patients compared to OA patients and healthy controls. Our results were in line with the study of Colin *et al* [47], which demonstrated increased percentages of IL-17+ and IL-22+CD4+ T cells in PBMCs from treatment-naïve patients with early RA. IL-22 production by Th17 cells has been demonstrated to be dependent on IL-23 [49], and elevated serum IL-23 level was detected in the patients with AS [20]. Our data suggest that the tendency to develop both CD4+IL-22+IL-17+IFNγ- T cells and Th1/Th17 cells may be a common immunologic characteristic shown in AS and RA patients.

In conclusion, our experiments showed that the frequencies of Th22 cells and Th17 cells were significantly increased in AS and RA patients compared to OA patients and healthy controls. Plasma IL-22 levels, which correlated positively with Th22 cells, were also demonstrated to be elevated in both AS and RA patients. The increasing percentages of Th22 cells and Th17 cells and the elevation of IL-22 may play important roles in the pathogenesis of AS and RA. Thus, Th22 cells and IL-22 as well as Th17 cells may prove to be a promising therapeutic targets for AS and RA. Further studies are awaited to clarify the pathophysiologic role of Th22 in AS and RA and determine the situation of Th22 cells in AS and RA patients’ joints.

## Supporting Information

Figure S1 **a, b and c,** No positive correlations were found between the percentage of Th22 cells and ESR, CRP as well as BASDAI in AS patients. d, e and f, No positive correlations were found between the percentage of Th17 cells and ESR, CRP as well as BASDAI in AS patients. **g, h and i,** No positive correlations were found between the levels of plasma IL-22 and ESR, CRP as well as BASDAI in AS patients. **j and k,** No positive correlations were found between the levels of plasma IL-22 and CRP as well as DAS28 in RA patients. **i,** No positive correlation was found between the levels of plasma IL-22 and the percentage of Th17 cells in RA patients.
(TIF)

Figure S2 The number of Th22 cells and Th17 cells in per volume of peripheral blood in AS patients, RA patients and healthy controls. **a,** The number of Th22 cells was significantly increased in AS and RA patients compared with healthy controls after stimulation with phorbol myristate acetate, ionomycin, and monensin for 4 h. **b,** The number of Th17 cells was significantly increased in AS and RA patients compared with healthy controls after stimulation with phorbol myristate acetate, ionomycin, and monensin for 4 h. (* = *P*<0.05)
(TIF)

Table S1 Major previous treatment of each patient of AS*
(DOC)

Table S2 Major previous treatment of each patient of RA*
(DOC)
