# Supplementary figures and images for: Increased Frequencies of Th22 Cells as well as Th17 Cells in the Peripheral Blood of Patients with Ankylosing Spondylitis and Rheumatoid Arthritis
Source: PLoS One. 2012 Apr 2;7(4):e31000. doi: 10.1371/journal.pone.0031000 (PMC3317658; doi:10.1371/journal.pone.0031000)

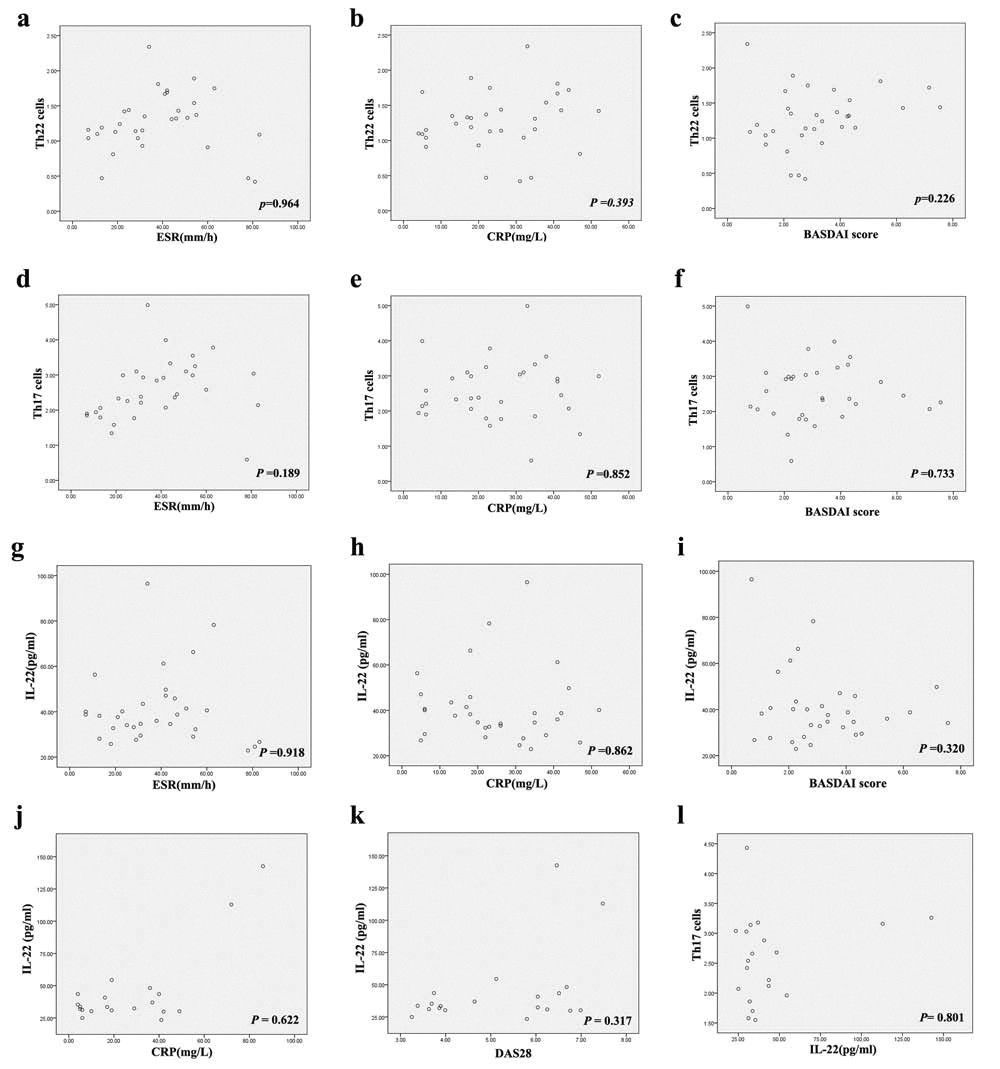

Supplement: Figure S1 — a, b and c, No positive correlations were found between the percentage of Th22 cells and ESR, CRP as well as BASDAI in AS patients. d, e and f, No positive correlations were found between the percentage of Th17 cells and ESR, CRP as well as BASDAI in AS patients. g, h and i, No positive correlations were found between the levels of plasma IL-22 and ESR, CRP as well as BASDAI in AS patients. j and k, No positive correlations were found between the levels of plasma IL-22 and CRP as well as DAS28 in RA patients. i, No positive correlation was found between the levels of plasma IL-22 and the percentage of Th17 cells in RA patients. (TIF) [file pone.0031000.s001.tif]

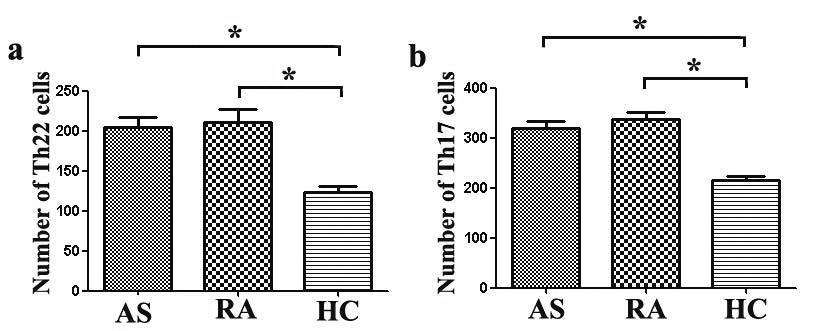

Supplement: Figure S2 — The number of Th22 cells and Th17 cells in per volume of peripheral blood in AS patients, RA patients and healthy controls. a, The number of Th22 cells was significantly increased in AS and RA patients compared with healthy controls after stimulation with phorbol myristate acetate, ionomycin, and monensin for 4 h. b, The number of Th17 cells was significantly increased in AS and RA patients compared with healthy controls after stimulation with phorbol myristate acetate, ionomycin, and monensin for 4 h. (* = P<0.05) (TIF) [file pone.0031000.s002.tif]
