# Supplementary material for: Increased Frequencies of Th22 Cells as well as Th17 Cells in the Peripheral Blood of Patients with Ankylosing Spondylitis and Rheumatoid Arthritis
Source: PLoS One. 2012 Apr 2;7(4):e31000. doi: 10.1371/journal.pone.0031000 (PMC3317658; doi:10.1371/journal.pone.0031000)
Supplement: Table S1 — Major previous treatment of each patient of AS* (DOC) [file pone.0031000.s003.doc]

**Table S1.** Major previous treatment of each patient of AS*****

| Patient/sex/age | Major previous Treatment | | | |
| --- | --- | --- | --- | --- |
|  | NSAIDs | DMARDs | Anti-TNF | Steroid |
| 1/ M /26 | + | - | - | - |
| 2/ M /28 | - | - | - | - |
| 3/ F /33 | + | - | - | - |
| 4/ M /23 | + | - | - | - |
| 5/ M /29 | + | - | Inflix. | - |
| 6/ M /22 | - | - | - | - |
| 7/ M /31 | + | - | - | - |
| 8/ F /38 | - | - | Etan. | - |
| 9/ M /43 | + | MTX | - | - |
| 10/ M /45 | - | - | Etan. | - |
| 11/ M /53 | + | - | - | Pred. |
| 12/ M /29 | + | - | - | - |
| 13/ M /32 | + | - | - | - |
| 14/ M /55 | + | SSZ | - | - |
| 15/ F /43 | + | MTX | - | - |
| 16/ M /61 | + | - | Etan. | - |
| 17/ M /25 | + | - | - | - |
| 18/ F/35 | + | MTX | - | - |
| 19/ M /52 | + | - | - | Pred. |
| 20/ M /38 | + | - | - | - |
| 21/ M /31 | + | - | - | - |
| 22/ M /42 | + | SSZ | - | Pred. |
| 23/ M /27 | + | - | - | - |
| 24/ M /39 | + | MTX | - | - |
| 25/ M /42 | - | - | Etan. | - |
| 26/ M /31 | + | SSZ | - | - |
| 27/ M /26 | + | - | - | - |
| 28/ M /38 | - | - | - | - |
| 29/ M /51 | - | - | Etan. | Pred. |
| 30/ M /36 | + | - | - | - |
| 31/ M /44 | + | MTX | Etan. | - |
| 32/ F /24 | + | - | - | - |

*****NSAIDs = nonsteroidal anti-inflammatory drugs; DMARDs = disease-modifying antirheumatic drugs; Anti-TNF = anti-tumor necrosis factor; MTX = methotrexate; Inflix. = infliximab; SSZ = sulfasalazine; Etan. = etanercept; Pred. = prednisone.
