# Supplementary material for: Increased Frequencies of Th22 Cells as well as Th17 Cells in the Peripheral Blood of Patients with Ankylosing Spondylitis and Rheumatoid Arthritis
Source: PLoS One. 2012 Apr 2;7(4):e31000. doi: 10.1371/journal.pone.0031000 (PMC3317658; doi:10.1371/journal.pone.0031000)
Supplement: Table S2 — Major previous treatment of each patient of RA* (DOC) [file pone.0031000.s004.doc]

**Table S2.** Major previous treatment of each patient of RA*****

| Patient/sex/age | Major previous Treatment | | | |
| --- | --- | --- | --- | --- |
|  | NSAIDs | DMARDs | Anti-TNF | Steroid |
| 1/ F /38 | + | MTX, SSZ | - | - |
| 2/ F /56 | + | MTX | - | - |
| 3/ F /47 | + | MTX | - | - |
| 4/ F /35 | + | MTX | Etan. | - |
| 5/ M /58 | + | MTX, SSZ | - | Pred. |
| 6/ F /40 | - | MTX, SSZ | - | - |
| 7/ F /37 | + | MTX | - | Pred. |
| 8/ F /47 | + | MTX | - | - |
| 9/ M /47 | + | MTX | - | - |
| 10/ F /54 | + | MTX | Etan. | - |
| 11/ F /44 | + | - | - | - |
| 12/ F /57 | + | MTX | Etan. | - |
| 13/ F /68 | + | MTX, SSZ | - | Pred. |
| 14/ M /53 | + | MTX | - | - |
| 15/ F /37 | + | MTX | - | - |
| 16/ F /49 | + | MTX, Lef. | Etan. | - |
| 17/ M /55 | + | MTX | - | - |
| 18/ F /53 | + | MTX | - | - |
| 19/ F /39 | - | MTX | - | Pred. |
| 20/ F /35 | + | MTX, Lef. | - | - |

*****NSAIDs = nonsteroidal anti-inflammatory drugs; DMARDs = disease-modifying antirheumatic drugs; Anti-TNF = anti-tumor necrosis factor; MTX = methotrexate; Lef. = leflonomide; SSZ = sulfasalazine; Etan. = etanercept; Pred. = prednisone.
